# Influence of Dental Composite Viscosity in Attachment Reproduction: An Experimental in Vitro Study

**DOI:** 10.3390/ma12234001

**Published:** 2019-12-02

**Authors:** Vincenzo D’Antò, Simone Muraglie, Beatrice Castellano, Ettore Candida, Maria Francesca Sfondrini, Andrea Scribante, Cristina Grippaudo

**Affiliations:** 1 Department of Neuroscience, Reproductive Science and Dentistry, University of Naples Federico II, 80138 Naples, Italy; 2 Dental Institute, Catholic University of the Sacred Heart, 00195 Rome, Italy; simone.muraglie01@icatt.it (S.M.); beatrice.castellano02@icatt.it (B.C.); ettore.candida@icatt.it (E.C.); 3 Unit of Orthodontics and Pediatric Dentistry - Section of Dentistry - Department of Clinical, Surgical, Diagnostic and Pediatric Sciences, University of Pavia, 27100 Pavia, Italy; francesca.sfondrini@unipv.it (M.F.S.); andrea.scribante@unipv.it (A.S.); 4 Head and Neck Department, Fondazione Policlinico Gemelli IRCCS, Dental Institute, Catholic University of Sacred Heart, 00195 Rome, Italy; cristina.grippaudo@unicatt.it

**Keywords:** orthodontics, attachments, composite resin, clear aligners, detail reproduction, viscosity degree

## Abstract

Background: Attachments are composite auxiliaries that are used during a clear aligner orthodontic therapy to achieve difficult tooth movements. Two important factors are the planned configuration and the actual position of these auxiliary elements to obtain the desired force system. The aim of the present study was to evaluate the role of different composite materials in the correct reproduction of attachment shape and position. Methods: The materials that were considered in the study were a flowable resin, a dental restorative material, and an orthodontic composite. The attachments were created on three models of extracted teeth. Once the impressions were performed, 25 attachments of different shapes were added onto each virtual model to obtain the necessary templates to make the attachments. Each tested material was used to create a set of 25 attachments that were then scanned with an accuracy of 10μm. The resultant STL (stereolithography) files were superimposed onto the ones from the initial virtual plan, through Geomagic software, and the aligned scans were then compared while using a color map. The parameters that were calculated to make a comparison between the created attachments and the ideal ones were the maximum deviation in defect and in excess, the overflow, and the volume’s difference. In addition to these measurements comparing the three above-mentioned groups, the difference in volume between all the ideal and realized samples were analyzed. To test for differences among the three groups, a one-way Analysis of variance (ANOVA) was used with a Bonferroni post-hoc test. The level of significance was set at *p* < 0.05. Results: No statistically significant results were found between the three groups regarding the maximum value in defect, the maximum value in excess, and the minimum value as control, while a statistically significant difference was found between the overflow of orthodontic resin when compared to the flowable composite. Conclusions: The three materials that were used in this study were appropriate for attachment fabrication. The fidelity of attachment reproduction was similar when using the three different composites. The orthodontic composite showed more overflow when compared with the flowable one.

## 1. Introduction

In recent decades, a significant increase in patient demand for aesthetic orthodontic appliances has been recorded. Therefore, aligner systems have developed progressively and different brands of clear aligners are now available since their introduction [1].

The possibility of managing severe malocclusions with the sequential application of these clear thermoplastic devices has already been debated [2]. Severely rotated or severely tipped teeth, open bites, extrusion, crowding, and spacing over 5 mm were described as the conditions that are difficult to treat with aligners [3]. Manufacturers began to provide new highly elastic aligner materials and they encouraged the use of auxiliary elements, such as resin attachments on tooth surfaces, in order to extend their clinical application and to improve their predictability [4].

These composite resin elements should be added to the tooth surface not only to enhance aligners retention, but also to achieve difficult tooth movements, all while maintaining the treatment’s aesthetic appearance [5]. For example, the absence of these supplementary elements could lead to undesired inclinations of the tooth during the translation movements [6].

The position and configuration of the attachment itself are the most important factors affecting the attachment’s efficiency and aligner fitting [7]. Furthermore, the type of composite resin used to create the attachments could be relevant and must meet specific requirements to ensure the validity of these auxiliary elements. The ideal dental composite resin must preserve its features over time, since it should stay in the patient oral cavity throughout the orthodontic treatment period and, more importantly, it must faithfully reproduce the attachment active surface, as it is strongly related to the force system delivered by the aligner [8].

Although the importance of the material selection in this context is clear, only a small number of researches have investigated which type of composite resin is the most suitable for this clinical application [8,9,10]. The same authors [8,9,10] highlighted that further studies are needed to investigate other properties, such as hardness, detail reproduction, bond strength, ease of use, and cost.

The most challenging step when placing attachments is to effectively filling the hollows of the template, and the planned position and shape may be influenced by material consistency and viscosity [11]. In this context, flowable composites are designed to be dispensed from ultrafine needles in narrow spaces, whereas the packable composite solidity enables material modeling with metallic instruments [12,13,14], and high viscosity composites might present the advantage of better stability.

Therefore, the aim of the present study was to evaluate whether the use of composites with different viscosities and consistencies might influence the shape and volume of attachments made on extracted teeth. 

The null hypothesis of our investigation is that there are no differences in the shape and volumes of planned and realized attachments while using composites with different viscosities.

## 2. Materials and Methods

### 2.1. Sample Size Evaluation

The sample size of the present study was calculated setting the power of study at 80% and the level of significance at 0.05. The effect size eta-squared is 0.37, while assuming a standard deviation of 1 for each group, while considering 25 attachments for each group and a total of 75 attachments. This can be considered to be a large effect size, and it was chosen because the goal of the study is to assess clinically significant differences.

Study materials:

Three composite resins were selected based on three different degrees of viscosity: a low-viscosity flowable resin (ENAMEL plus HRi® Flow HF, GDF GmbH), a medium-viscosity orthodontic composite (Bracepaste® Medium Viscosity Adhesive, AO), and a high -viscosity dental restorative material (ENAMEL plus HRi® Enamel, GDF GmbH) (Table 1).

We applied the three different materials to three pairs of phantom dental arches to test their effectiveness when reproducing the attachments. Each pair received 25 attachments on the vestibular or lingual surface of the teeth, according to the sample size calculation. In this way, it was possible to test one type of composite for each pair of dental arch, thus reproducing the real operative procedures.

### 2.2. Operative Procedures

Three phantom models of patients’ dental arches were made while using extracted teeth.

Briefly, the teeth were chosen depending on the following inclusion criteria: well-preserved dental crown, extraction for orthodontic, periodontal, or prosthetic reasons. Exclusion criteria were: presence of carious lesions, prosthetic crowns, or even fillings of any material.

All of the teeth were stored in a physiological solution after extraction, and then divided into four morphological groups (incisors, canines, bicuspids, and molars) and used to create realistic upper and lower arches of the three phantom models. Once the models were created, dental impressions using polyvinyl siloxane material (Flextime Heavy Tray/Correct Flow, Kulzer GmbH, Wasserburg, Germany) were taken and sent to the manufacturer of the aligners (Airnivol S.r.l., Pisa, Italy) in efforts to fabricate the templates for attachment placement. Impression records and frontal, lateral occlusal photographs were also sent as usually required by aligner manufacturers. 

**Figure 1:**
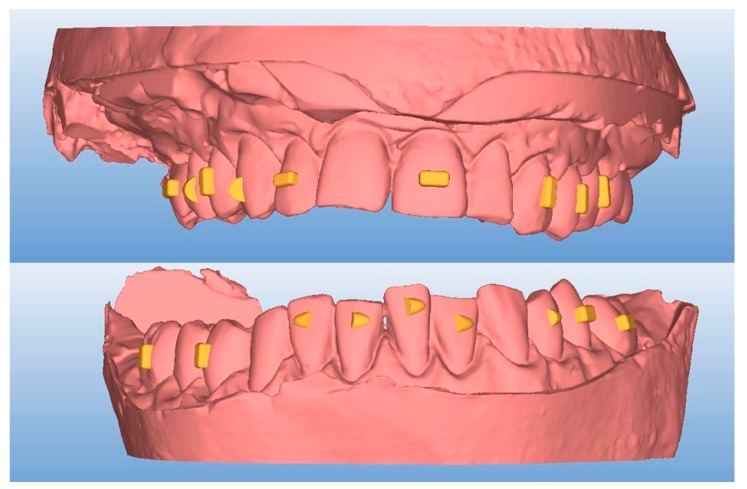
Projected attachments after PVS (polyvinyl siloxane) records scan (vestibular vision).

Thereafter, the desired attachment shapes and positions were selected while using the AirCheck tool that was provided by the aligner manufacturers (Figure 1).

Table 2 describes the description and details of the requested attachments.

For each phantom model, 13 attachments for the upper arch and 12 for the lower arch were requested. Once the templates, including the appropriate attachments’ hollows, were received, it was possible to proceed with the attachment placement while using three different composite resins on the phantom models, as previously described [9]. Briefly, the workflow of attachments placing went as follows:

Step 1. Enamel pretreatment: To commence, the vestibular tooth surface, where the attachment needed to be placed, was etched while using a 37% orthophosphoric acid (Scientific Pharmaceuticals, Inc., Pomona, CA, USA) for 30 seconds, rinsed with plenty of water, and then dried with light air.

Step 2. Bonding: a thin bonding agent layer (iBOND Total Etch, Heraeus Kulzer, Hanau, Germany) was then applied and light-cured for 20 seconds while using a high power lamp (DB685 Super Dual, COXO Dental, Foshang, China).

Step 3. Composite loading: The resin composite was loaded into each hollows of the attachment template by using a thin tip syringe for low and medium viscosity materials. Otherwise, high viscosity resin was loaded with a metallic spatula. Once the composite was loaded, it was fully situated onto the teeth and gentle pressure around each attachment was then applied with tweezers.

**Figure 2:**
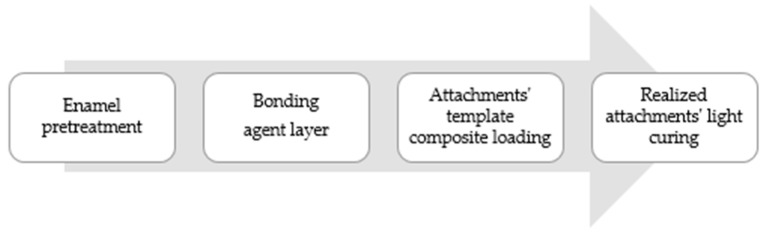
Attachments clinical technique bonding phases.

Step 4. Light curing: The composite resin was light-cured according to the composite instructions, selecting the most effective wavelength (400–500 nm). No polishing or finishing procedure was done once the attachment template was removed (Figure 2).

For this study, three materials were selected with three different levels of viscosity in order to test composites with different viscosities and consistencies: a low-viscosity flowable resin (ENAMEL plus HRi® Flow HF, GDF GmbH), a medium-viscosity orthodontic composite (Bracepaste® Medium Viscosity Adhesive, AO), and a high -viscosity dental restorative material (ENAMEL plus HRi® Enamel, GDF GmbH) (Table 1). Each step was repeated for each composite resin selected by using one phantom model per time.

### 2.3. 3D Analysis

Once the attachments were placed, the upper and lower arches of the phantom models were scanned while using a laser light three-dimensional (3D) scanner (D800, 3Shape, Copenaghen, Denmark), with a declared accuracy of 10 μm, and converted in a 3D mesh model (stl). The obtained 3D files of the phantom models were analyzed while using a reverse engineering analysis software (Geomagic Control, 3DS Systems, Rock Hill, South Carolina, USA) by following the previously described protocols to compare the shape between the realized and programmed attachments [15,16]. Briefly, the obtained 3D models were superimposed with the ones that were derived from the virtual plan, using a surface-based superimposition. The landmark areas that were selected to achieve the superimposition process were the palatal area and the occlusal teeth surfaces.

Once the models were superimposed, the virtual planning model was used as a reference and a surface deviation analysis was carried out. The software automatically calculated the linear distances (Euclidean distances) between 100% of the surface of the two superimposed models and then provided a color map that highlighted the lack (blue color) and the excess (red/orange color) areas inside and outside the perimeter of the attachment shape.

For this study, the maximum deviation evaluated was set to ±2 mm and the tolerance range (green color) within the deviation values considered acceptable was set to ±0.1 mm.

**Figure 3:**
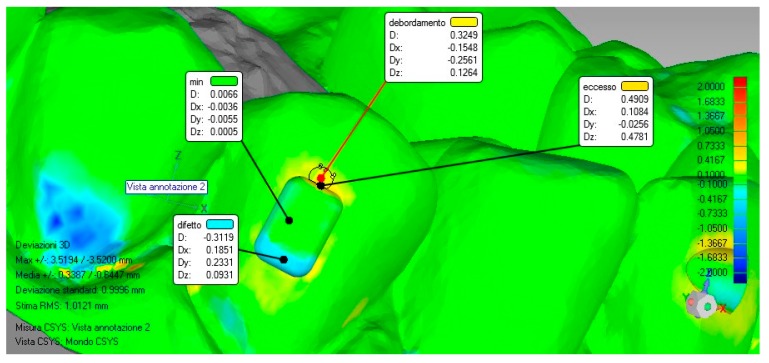
Analyzed parameters. Maximum value in defect (blue), maximum value in excess (red), overflow, and minimum value (green) as control.

Measurements were carried out at the level of the attachment’s surface to distinguish the maximum value in defect and in excess, inside the perimeter of the attachment shape, and the overflow value outside that perimeter, once this procedure was done for each model; moreover, a minimum value was detected as a control mechanism to consider the accuracy of the alignment between the files (Figure 3).

**Figure 4:**
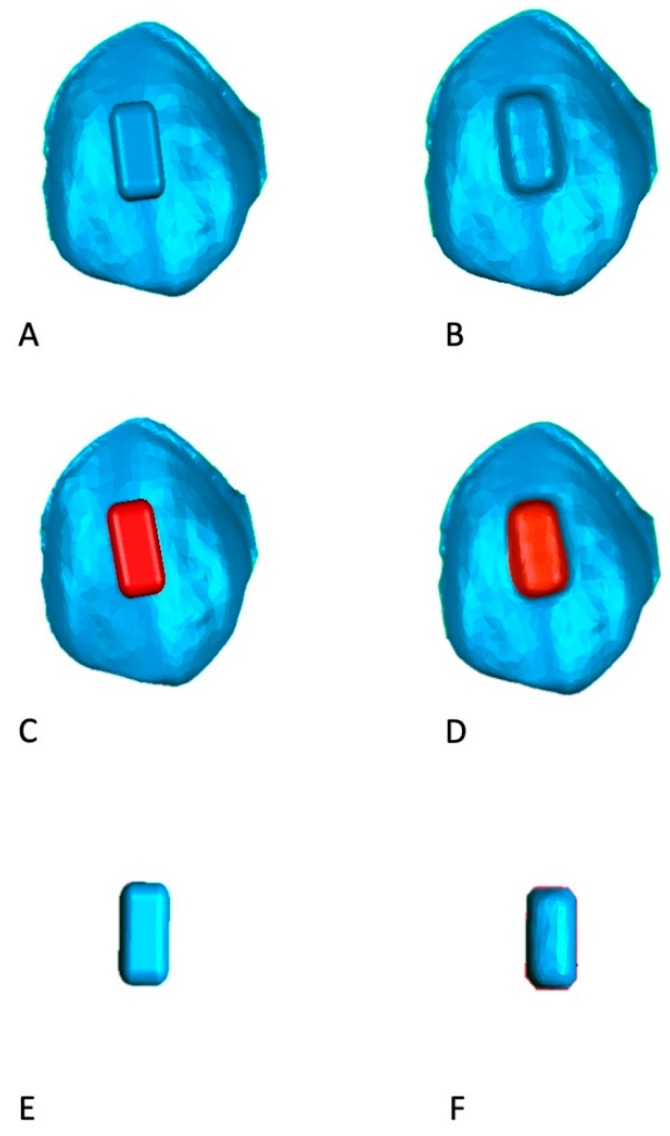
Comparison between programmed and real attachment’s volumes. The ideal (**A**) and real (**B**) attachments are first segmented (**C**,**D**) and then the volumes (**E**,**F**) are calculated while using a specific function of Geomagic Contol software.

Furthermore, volumetric measurements of auxiliary elements were carried out through Geomagic software (Figure 4) by segmenting the real and ideal attachments and while using a specific tool of the software in order to assess whether any differences in volumes were present between programmed and realized attachments.

### 2.4. Methodological Error

The same operator repeated the landmark selection and the measurements of each parameter after two weeks to establish intra-operator reliability. For all measurements, Dahlberg’s formula [17] was used to calculate the standard error on the repeated sets of measurements. Bland–Altman plots were used to check for the intra-observer reliability between the two sets of measurements. 

### 2.5. Statistical Analysis

Statistical tests were performed by SPSS (Statistical Package for the Social Sciences) software (version 20.0, IBM Corp., New York, NY, USA) to assess the central tendencies and dispersion measurements of the three groups (flow, composite, orthodontic) for each observed parameter. Such data were then represented through boxplots. A one-way ANOVA was used to verify the difference among the three groups. Wherever a statistical difference was identified, a Post-Hoc test was performed. Finally, a T-test was conducted to compare the whole ideal and real attachment volume values.

## 3. Results

**Figure 5:**
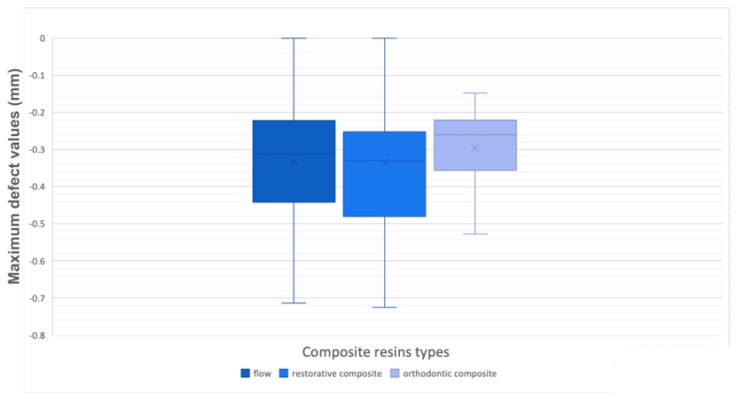
Box-plots of maximum defect values.

**Figure 6:**
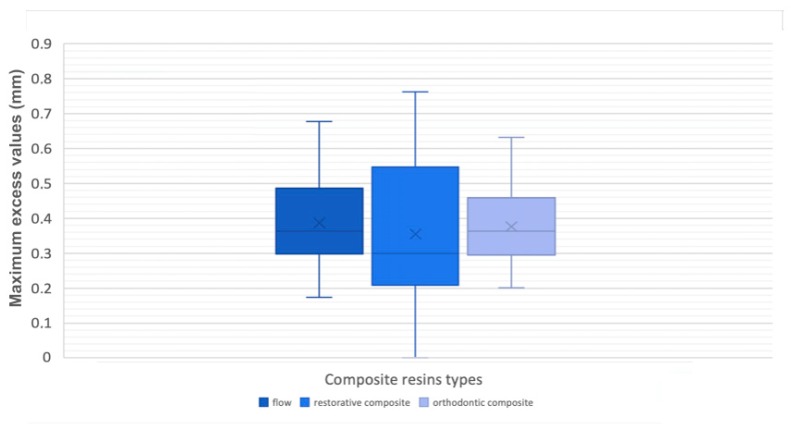
Box-plots of maximum excess values.

Regarding the error of the method, the standard error was 0.22 mm for the maximum excess, 0.11 mm for the maximum defect, 0.12 mm for the overflow, 0.43 mm^2^ for the ideal volume, and 0.72 mm^2^ for the real volume. Bland–Altman plots revealed no systematic errors, confirming the intra-observer reliability of the measurements. When analyzing the maximum excess and defect deviation into the attachments’ perimeters of the three composite resin groups, boxplots were generated to assess data normality (Figure 5 and Figure 6, Table 3 and Table 4).

It was decided that the existing outliers should be eliminated by watching the resultant graphics. Thereafter, for the two parameters, the one-way ANOVA was carried out and a non-statistically significant outcome, namely an irrelevant difference, was found (0.470, 0.583; *p* > 0.05) between the flow, the dental restorative composite, and the orthodontic composite (Table 5 and Table 6).

**Figure 7:**
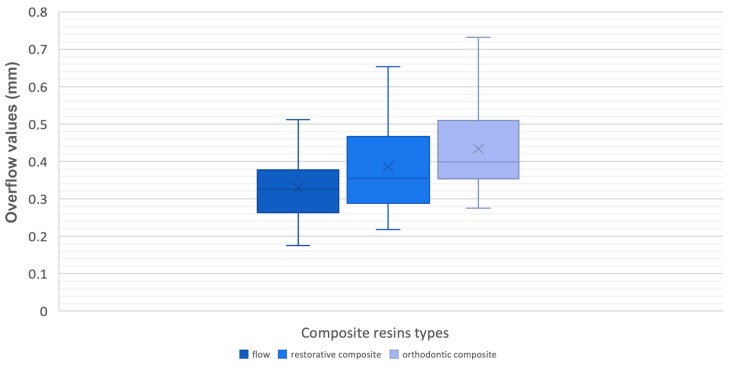
Box-plots of overflow values.

The same was for the overflow. A statistically significant difference between the groups was shown once the outliers were removed from the ANOVA analysis (*p* < 0.5), with a significance level of 0.026 (Figure 7, Table 7 and Table 8).

The Bonferroni Post-Hoc test was used to investigate the obtained result. A statistically relevant difference between the flowable and orthodontic composites was found, with a significance level of 0.021, for a 95% confidence interval with a lower limit of 0.0107 and an upper limit of 0.1752 (Table 9).

**Figure 8:**
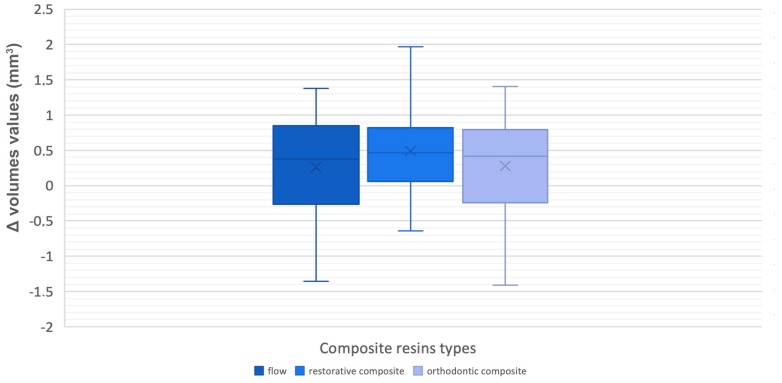
Box-plots of ∆ volumes values.

Therefore, the discrepancy was recorded between materials with low and medium viscosity degrees. When compared to low viscosity composites, a medium viscosity composite predisposes to higher overflow values. The last analysed parameter among the three groups was the difference in the volume values between the programmed and the realized attachments. A non-statistically significant outcome was found (0.433; *p* > 0.05) (Figure 8; Table 10 and Table 11).

**Figure 9:**
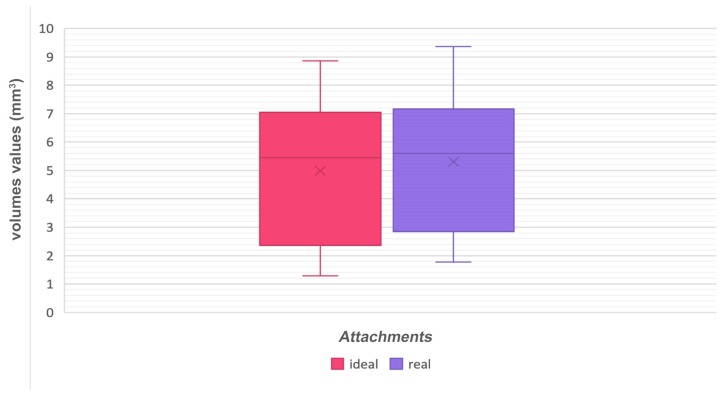
Box-plots comparing ideal and real volumes.

Even the T-test between the whole ideal and real attachments’ volume values gave a non-statistically significant result (0.515; *p* > 0.05) (Figure 9; Table 12 and Table 13). 

## 4. Discussion

Some clear aligner systems require bonded resin attachments to enhance aligner retention and allow more complex tooth movements [1]. Correct attachment use can significantly influence treatment predictability, as Garino et al. [18] demonstrated from the analysis of the composite attachments’ effectiveness in controlling upper-molar movement with aligners. On the other hand, attachments may have different effectiveness, depending on their shape [19].

Some attachments’ characteristics depend on their own material composition. As described in the literature, a different resin composite might have different performance in terms of translucency, stain resistance, and hardness. In this context, Feinberg et al. [10] analyzed the translucency, stain resistance, and hardness of composites used for Invisalign attachments, testing two dental restorative composites and three orthodontic adhesives with different properties in filler content. The material ability to prevent shape and surface alteration during six months of treatment was also evaluated by Barreda et al. [9], demonstrating that the use of different composites could affect the surface, but not the shape of the attachments.

As shown in Table 1, the materials that were investigated in the present study represent three different categories: flowable, orthodontic, and restorative composites, each with a different percentage in filler content (70%, 72%, and 80% respectively). Low viscosity composites are usually suitable for small cavities and dental sealants [20]. On the other hand, high viscosity composites are used in conservative dentistry for dentin and enamel restoration [21]. Intermediate viscosity materials are requested for orthodontic bracket bonding [22,23]. Nowadays, there are no guidelines regarding the ideal viscosity of orthodontic attachments during clear aligner therapy. As the thickness of the aligner is not altered during ten-day use [24], the fitting of the aligner could be mainly altered by attachment composition [8], thus influencing the effectiveness of dental movement [25].

The results of our investigation demonstrated that there were no differences while analyzing the shape and volumes of planned and realized attachments using three composites of different viscosity, while a significant difference has been found for the overflow parameter. In particular, the orthodontic composite (Bracepaste® Medium Viscosity Adhesive, AO), with a medium viscosity degree, showed greater overflow tendency when compared to the flowable composite (ENAMEL plus HRi® Flow HF, GDF GmbH), with a low viscosity degree. Furthermore, the findings for each composite used showed an equivalent level of accuracy when comparing each viscosity group while, comparing the volumes of the attachments that were obtained with the ones programmed on the virtual plan, it is possible to state that the configuration is properly reproduced by all of the tested composite resins and no differences were found between the groups. 

This study presents some limitations, because it does not consider the complexity of the in vivo procedure and it is restricted to only three resin materials.

## 5. Conclusions

The results of this study demonstrated that the use of different composites with different viscosities does not influence the shape and volume of attachments reproduced with a template on extracted teeth. Furthermore, the orthodontic composite showed more overflow respect when compared with to the flowable one.

## Figures and Tables

**Table 1:** Description and composition of the tested materials.

Commercial Name	Composite Classification	Viscosity	Resin Matrix	Filler Weight	Filler Composition
ENAMEL plus HRi® Flow HF	Flowable	low	Urethane Dimethacrylate-Butanediol dimethacrylate	70%	Glass (4.3 micron and 0.7 micron)-Silica (0.04 micron)
Bracepaste®	Orthodontic composite	medium	Bis GMA and Bis EMA	72%	silanized strontium aluminum boron silicate glass and silanized silica
ENAMEL plus HRi® Enamel	Homogeneous microhybrid	high	Diurethandimethacrylate–bisGMA -1,4-butandioldimethacrylate	80%	nano zirconium oxide (20 nanometer)-glass (1 micron)

**Table 2:** Attachments’ shapes and positions.

Upper Arch		Lower Arch	
Tooth	Position	Tooth	Position
16	horizontal for retention	36	horizontal for retention
15	optimized for rotation	35	horizontal for retention
14	rectangular for rotation	34	optimized for rotation
13	optimized for rotation	32	vestibular optimized for rotation
12	vestibular for extrusion	32	lingual optimized for rotation
11	palatal for extrusion	31	vestibular optimized for rotation
21	vestibular for extrusion	31	lingual optimized for rotation
22	palatal for extrusion	41	vestibular optimized for rotation
23	rectangular for distalization	41	lingual optimized for rotation
24	vertical rectangular	42	vestibular optimized for rotation
25	vertical rectangular	42	lingual optimized for rotation
26	palatal rectangular for rotation	44	vertical rectangular
-	-	46	vertical rectangular

**Table 3:** Descriptive statistics for defect values (mm).

	95% Confidence Interval of Mean							
Material	N	Mean	Std.Deviation	Std. Error	Lower Bound	Upper Bound	Minimum	Maximum
Flow	25	−0.3337	0.15767	0.03153	−0.3988	−0.2666	−0.71	0.00
Restorative	25	−0.3341	0.19976	0.03995	−0.4166	−0.2516	−0.72	0.00
Orthodontic	24	−0.2841	0.11705	0.02389	−0.3336	−0.2347	−0.53	0.00
Total	74	−0.3178	0.16174	0.01880	−0.3552	−0.2803	−0.72	0.00

**Table 4:** Descriptive statistics for excess values (mm).

	95% Confidence Interval of Mean							
Material	N	Mean	Std.Deviation	Std. Error	Lower Bound	Upper Bound	Minimum	Maximum
Flow	23	0.4091	0.16625	0.03467	0.3372	0.4810	0.17	0.90
Restorative	25	0.3551	0.23194	0.04639	0.2594	0.4509	0.00	0.76
Orthodontic	22	0.3764	0.11347	0.02419	0.3261	0.4267	0.20	0.63
Total	70	0.3795	0.17876	0.02137	0.3369	0.4222	0.00	0.90

**Table 5:** One-way ANOVA for defect values.

Comparison	Sum of Squares	df (degrees of freedom)	Mean Square	F	Sig.
Between groups	0.040	2	0.020	0.762	0.470
Within groups	1.869	71	0.026	-	-
Total	1.910	73	-	-	-

**Table 6:** One-way ANOVA for excess values.

Comparison	Sum of Squares	df	Mean Square	F	Sig.
Between groups	0.035	2	0.018	0.544	0.583
Within groups	2.170	67	0.032	-	-
Total	2.205	69	-	-	-

**Table 7:** Descriptive statistics for overflow.

	95% Confidence Interval of Mean							
Material	N	Mean	Std.Deviation	Std. Error	Lower Bound	Upper Bound	Minimum	Maximum
Flow	23	0.3408	0.10008	0.02087	0.2975	0.3841	0.17	0.58
Restorative	24	0.3853	0.11811	0.02411	0.3354	0.4352	0.22	0.65
Orthodontic	23	0.4338	0.12107	0.02524	0.3814	0.4861	0.27	0.73
Total	70	0.3866	0.11814	0.01412	0.3584	0.4148	0.17	0.73

**Table 8:** One-way ANOVA for overflow values.

Comparison	Sum of Squares	df	Mean Square	F	Sig.
Between groups	0.099	2	0.050	3.857	0.026
Within groups	0.864	67	0.013	-	-
Total	0.963	69	-	-	-

**Table 9:** Bonferroni test multiple comparisons. Dependent Variable: Overflow. * The mean difference is significant at the 0.5 level.

Material					95% Confidence Interval	
	(J) Groups	Mean Difference (I-J)	Std. Error	Sig.	Lower Bound	Upper Bound
Flow	Restorative	−0.04446	0.03313	0.552	−0.1258	0.0369
	Orthodontic	−0.09296 *	0.03348	0.021	−0.1752	−0.0107
Restorative	Flow	0.04446	0.03313	0.552	−0.0369	0.1258
	Orthodontic	−0.04850	0.03313	0.444	−0.1298	0.0329
Orthodontic	Flow	0.09296	0.03348	0.021	0.107	0.1752
	Restorative	0.04850	0.03313	0.444	−0.0329	0.1298

**Table 10:** Descriptive statistics (mm^2^) for volumes.

Material					95% Confidence Interval of Mean			
	N	Mean	Std.Deviation	Std. Error	Lower Bound	Upper Bound	Minimum	Maximum
Flow	25	0.2608	0.74551	0.14910	−0.0469	0.5686	−1.36	1.38
Restorative	24	0.4937	0.65681	0.13407	0.2163	0.7710	−0.64	1.97
Orthodontic	25	0.2791	0.70524	0.14105	−0.0120	0.5703	−1.41	1.41
Total	74	0.3425	0.70238	0.08165	0.1798	0.5053	−1.41	1.97

**Table 11:** One-way ANOVA for volume values.

Comparison	Sum of Squares	df	Mean Square	F	Sig.
Between groups	0.815	2	0.408	0.822	0.443
Within groups	35.198	71	0.496	-	-
Total	36.013	73	-	-	-

**Table 12:** Independent samples test comparing the ideal and real volumes.

		Levene’s Test for Equality of Variance		T-Test for Equality of Means						
									95% Confidence Interval of the Difference	
		F	Sig.	t	df	Sig. (2-tailed)	Mean Difference	Std. Error Difference	Lower	Upper
Volumes	Equal variances assumed	0.425	0.515	−0.866	148	0.388	−0.32216	0.37179	−1.05686	0.41255
	Equal variances not assumed	-	-	−0.866	147.789	0.388	−0.32216	0.37179	−1.05687	0.41256

**Table 13:** Group statistics for ideal and real volumes.

	Groups	N	Mean	Std. Deviation	Std. Error Mean
**Volumes**	Ideal	75	4.9879	2.31939	0.26782
	Real	75	5.3100	2.23332	0.25788
